# Questioning the questions: Methods used by medical schools to review internal assessment items

**DOI:** 10.15694/mep.2021.000037.1

**Published:** 2021-02-05

**Authors:** Bindu Menon, Jolene Miller, Lori M. DeShetler

**Affiliations:** 1 The University of Toledo

**Keywords:** Medical education, Assessment, Test item review

## Abstract

This article was migrated. The article was marked as recommended.

**Objective:** Review of assessment questions to ensure quality is critical to properly assess student performance. The purpose of this study was to identify processes used by medical schools to review questions used in internal assessments.

**Methods:** The authors recruited professionals involved with the writing and/or review of questions for their medical school’s internal assessments to participate in this study. The survey was administered electronically via an anonymous link, and participation was solicited through the DR-ED listserv, an electronic discussion group for medical educators. Responses were collected over a two-week period, and one reminder was sent to increase the response rate. The instrument was comprised of one demographic question, two closed-ended questions, and two open-ended questions.

**Results:** Thirty-nine respondents completed the survey in which 22 provided the name of their institution/medical school. Of those who self-identified, no two respondents appeared to be from the same institution, and participants represented institutions from across the United States with two from other countries. The majority (n=32, 82%) of respondents indicated they had a process to review student assessment questions. Most participants reported that faculty and course/block directors had responsibility for review of assessment questions, while some indicated they had a committee or group of faculty who was responsible for review. Most focused equally on content/accuracy, formatting, and grammar as reported. Over 81% (n=22) of respondents indicated they used NBME resources to guide review, and less than 19% (n=5) utilized internally developed writing guides.

**Conclusions:** Results of this study identified that medical schools are using a wide range of item review strategies and use a variety of tools to guide their review. These results will give insight to other medical schools who do not have processes in place to review assessment questions or who are looking to expand upon current procedures.

## Introduction

It is widely acknowledged that well-designed assessments positively impact student learning and drive the robust growth of a curriculum by identifying curricular strengths and weaknesses ( Norcini *et al.*, 2011). Medical schools have long recognized and emphasized the importance of internal examinations in ensuring that the graduating students are equipped with the knowledge and skills required to be competent and safe medical practitioners ( Miller, 1990). Well-written tests benefit both students and faculty. They motivate student learning and provide students with accurate performance feedback. These tests benefit faculty by providing feedback on teaching effectiveness. Conversely, the detrimental effects of poor item quality have also been well recognized by Downing (2005) and Tarrant and Ware (2008). Past research ( Downing, 2005; Jozefowicz *et al.*, 2002; Rodriguez-Diez *et al.*, 2016) has shown that multiple-choice questions often contain flaws that contribute to measurement error. Item-writing flaws have been shown to lead to construct-irrelevant variance thereby affecting the pass-fail outcomes for students in previous studies Downing, 2005; Downing, 2002). Generating quality assessments with well-written items on a regular basis has been reported a challenge by several medical schools according to Case, Holtzman and Ripkey (2001) and Pinjani, Umer and Sadaf (2015).

During the 2018-19 academic year, medical student feedback from course evaluations at our institution consistently identified issues with internal assessments. The identified problems included typographical, grammatical, and formatting errors as well as unclear question stems. Faculty were responsible for writing assessment questions, and course directors were charged with developing the assessments, but our medical school did not have a systematic process in place to review each assessment question prior to use in internal examinations. In the fall of 2019, college leadership established an item review committee to address student concerns by establishing a process for peer and editorial review of assessment items. Membership on this committee included faculty representing different areas of expertise: item writing, assessment, content, and editing. Soon after convening, the committee recognized the need for not only review of each assessment question, but also the need for a guide to aid faculty and directors in writing quality assessments. During committee review, members check each question’s formatting, grammar, and structure. If issues about the content of the item, such as questionable accuracy or confusing presentation, are identified, the course director is notified.

Peer-review of assessment questions for writing flaws is an effective way to improve question quality and performance ( Abozaid, Park and Tekian, 2017; Malua-Aduli and Zimitat, 2012; Wallach *et al.*, 2006). To assist the committee in its work, we were interested in how other medical schools reviewed assessment questions but were unable to find any research regarding the issue. The purpose of this study was to determine what processes, if any, medical schools use to review test items before the items are used on student assessments. We specifically sought to understand which individuals and groups were involved in review processes and what they included in their review. This purpose was achieved by answering the following research question: What methods do medical schools use to review questions that will be used to assess students’ knowledge and competence in internal examinations?

## Methods

### Design

We used a descriptive study with an online questionnaire to identify if medical schools have processes to review assessment items and to determine what methods they use in the review of questions. The Assessment Item Review survey (Supplementary File 1) consisted of one demographic question, two closed-ended questions, and two open-ended questions. The research was reviewed by The University of Toledo Social, Behavioral, and Educational Institutional Review Board and was found that the study did not meet the definition of human subjects’ research as outlined in 45 CFR 46.102(e)(1), and therefore did not require Institutional Review Board oversight or approval. We recruited medical school professionals into the study by email during spring 2020. The purpose of this study was explained, and participants were provided with an anonymous link to take the survey. Completion of the survey constituted informed consent.

### Sample

The sample was solicited from professionals subscribed to the DR-ED listserv, an electronic discussion group for medical educators. This email distribution list was selected because the membership includes medical school professionals who are involved with student assessment.

### Outcome measures

The survey contained an optional demographic question in which participants were asked to provide their institution/name of medical school. Two closed-ended questions followed. Participants were asked to indicate whether they had a process to review student assessment questions before they are used. If “No” were selected, the respondent was taken to the last question in the survey. The second closed-ended item asked participants to select which people or groups review student assessment questions before they are used, and what aspect(s) of questions they review. Respondents could select all that apply. Options for individuals and groups included Faculty member writing the question, Group of faculty members teaching related topics, Unit (course/block) director(s), Non-faculty academic staff/coordinator(s), Assessment question review committee, Curriculum committee, and Other. For the aspects of questions each individual/group reviews, response options were Content/accuracy, Item formatting, Grammar/spelling, and Other.

Two open-ended questions followed. Respondents were asked to list all sources and documents their medical school uses to guide student assessment question review (e.g., National Board of Medical Examiners [NBME] item writing manual, internally developed writing guide, NBME laboratory values). The last question of the survey prompted participants to share any other useful information regarding their medical school’s assessment question review process.

### Analysis

The analysis involved comparing the self-identified respondents’ institution to determine the possibility of duplication of responses from the same medical school. Next, tallies were run for the first closed-ended question to calculate the percentage of respondents who had a process for reviewing assessment items. In the second closed-ended question, we analyzed the frequency of people and groups that were selected for reviewing assessment questions, and the frequency for the type of review was analyzed to understand the roles of the people and groups tasked with reviewing assessment questions.

The second part of the analysis included coding of the qualitative responses. From the first open-ended question pertaining to sources and documents that the participants’ medical school uses to guide assessment question review, we grouped common terms and ranked sources from most to least cited. A qualitative analysis was also conducted on the last question regarding other useful information that participants chose to share, and themes were created based on their responses. A frequency threshold of 15% was utilized for identifying themes in the open-ended responses.

## Results/Analysis

A total of 39 participants completed the survey. Of this total, 22 provided the name of their institution/medical school. For those who self-identified, no two respondents appeared to be from the same institution, and participants represented schools from across the United States with two from other countries. All 39 participants answered the question about whether their school had a process to review student assessment questions. Just over 82% (n=32) reported that their medical school did have a process.

Table 1 shows the frequency of which person or group reviews assessment questions at the participants’ medical school, and of which aspect(s) the review consists (e.g., grammar/spelling).

**Table 1.** Person or group who reviews student assessment questions and aspects of the review

Person or Group	Aspect of Review			
	Content/Accuracy(number of respondents)	Item Formatting(number of respondents)	Grammar/Spelling(number of respondents)	Other(number of respondents)
Faculty member writing the question	26	18	20	4
Group of faculty members teaching related topics	11	10	9	1
Unit (course/block) director(s)	21	21	20	6
Non-faculty academic staff/coordinator(s)	0	9	10	2
Assessment question review committee	12	13	13	5
Curriculum committee	2	0	0	0
Other (list) ^a^	2	3	3	1

^a^ Medical Education Center, Academic Deans, Director of Assessment/Assistant Dean of assessment

The most common response (n=26) was that the faculty member writing the question holds responsibility for the content/accuracy of the assessment question. The next highest frequency (n=21) was unit (course/block) directors for the review of both content/accuracy and item formatting. Close behind, 20 participants indicated that the faculty member writing the question reviews for grammar/spelling, and 20 also reported that the unit directors review grammar/spelling. Less than half (n=18, 46%) of the respondents indicated that the faculty member writing the question at their medical school was responsible for item formatting. All other frequencies for the remaining choices of people and groups by review task were one-third or less. From these results, faculty and unit directors shared the highest frequency for review of assessment questions followed by assessment question review committees.

The type of question review was evenly dispersed among content/accuracy, item formatting, and grammar/spelling. The “Other” category was rarely chosen. The task of reviewing for content/accuracy was reported most (n=26) for the faculty member writing the question. Respondents indicated that item formatting was most carried out by unit directors (n=21). Meanwhile, grammar/spelling was selected as the responsibility of both faculty members writing the question and unit directors by 20 participants. Nine respondents indicated that non-faculty academic staff/coordinators reviewed item formatting, and 10 reported that they reviewed grammar/spelling; however, none of the participants selected content/accuracy for non-faculty academic staff/coordinators. By question review task, the faculty member writing the question and unit directors were the highest frequency. Only two medical schools indicated that their curriculum committee was involved with the question review process.

Twenty-seven participants provided sources and documents that their medical school uses to guide student assessment question review. The majority (n=21, 78%) of respondents listed the NBME item writing guide as a source they use to guide question review. Almost a third (n=8, 30%) of participants included NBME laboratory values as a document they utilize in the review process. Internally developed writing guides and item writing courses/workshops were each listed by five respondents.

Additional comments were provided by 20 participants. Three responses centered on the implication for faculty training to facilitate item review. For example, one respondent stated, “It is very important that teachers take a training course in learning assessment.” Another indicated that item review is best handled by course faculty, but individual faculty may view the process as a “waste of time.”

There were six comments regarding the quality of test questions. One participant explained, “Having a quality item bank software and good quality items that were peer reviewed before they were permitted to be used...were really important.” Another respondent described his/her review process in which item quality is reviewed and verified to confirm the quality of questions. Some who discussed the quality also included terms for validating their questions.

A third theme that emerged from 30% (n=6) of the comments was related to the roles of block/course directors in test item review. One participant stated that they have three levels of review, one of which includes the course director. Similarly, another participant said, “We have assessment vetting sessions by block directors.” Another indicated that following exam item review, suggestions are provided to course directors who then share feedback with the faculty.

Over one-third (n=7) of the comments focused on test item performance. Various respondents provided information regarding how their medical school tracks and uses item performance. For example, one respondent stated, “The performance statistics are used to update/improve question stems and answer choices.” Likewise, another said, “We track item performance before/after committee review.” Others noted tracking item performance over time or using statistical analytics for quality improvement. It should be noted that of the seven who indicated that they did not have a review process prior to items being used on an assessment, two shared that they analyzed item performance statistics after items are used.

Lastly, 75% (n=15) of respondents provided comments on the responsibility for test question review. Two respondents discussed a team approach, while another indicated that his/her medical school utilizes a peer review process. One participant said, “questions are viewed by at least two other faculty.” As mentioned previously, several made references to block directors, who held responsibility for item review at their schools. It appeared that some institutions split the responsibility of test question review among multiple groups (e.g., Assessment Office, Item Review Committee, and Course Director), and one had different processes depending on the medical student year (MD1 versus MD2).

## Discussion

Most of the participating medical schools had a process to review assessment questions before they are used on examinations. The responsibility for and the focus of the review differed by institution. We found that faculty and directors were most often responsible for the review of assessment questions based on these data. Assessment question review committees, while established at some of the respondents’ medical schools, were not as commonly reported as oversight for the review process as these individuals. In fact, it can be inferred from the data that only one-third of respondents had an assessment question review committee.

Because of the importance of internal examinations to assess student knowledge and competence, the greatest concern with poorly written items is construct-irrelevant variance. This is variance in examination scores that has nothing to do with student knowledge and competence. While there are a number of factors that contribute to this variance ( Downing, 2002), technical flaws in items contribute to irrelevant difficulty and testwiseness ( Paniagua and Swygert, 2016). Although examination questions are expected to vary in difficulty, that difficulty should be based on the content being assessed, not the structure of the question. The NBME has highlighted issues that contribute to irrelevant difficulty such as numerical responses presented in an illogical order, and the response option “None of the above.” Irrelevant difficulty introduces measurement error that decreases student scores, while testwiseness increases the scores for students who know how to take tests. These sorts of flaws include grammatical or logical cues (allowing the testwise student to rule out one or more options) and correct responses that are different in terms of length and detail ( Paniagua and Swygert, 2016).

The item review committee members in our medical school soon became cognizant of the fact that in ensuring test quality, the ultimate onus is on the faculty who are also the content experts, with the committee providing a more editorial review. Developing valid and reliable test items without construct-irrelevant variance is a critical skill for the faculty to hone. It seems institutions are giving more attention to faculty development to improve the quality of their exams ( Jozefowicz *et al.*, 2002; Abdulghani *et al.*, 2015; AlFaris *et al.*, 2015; Iramaneerat, 2012; Naeem, van der Vleuten and AlFaris, 2012), as studies have shown that faculty development and providing training in exam item writing improves the process of item writing and quality of exams ( Naeem, van der Vleuten and AlFaris, 2012; Tunk, 2001; Kim *et al.*, 2010). The importance of faculty development was reflected in respondents’ comments.

In institutions where individual faculty are solely responsible for the quality of assessment items, the use of performance analytics could be one way of tracking student progress and reviewing item performance. Yet, it is ideal to assign oversight of the items to a committee or director to ensure the overall quality of the exam, particularly in areas such as grammar and formatting. The establishment of an item review committee in our medical school that oversees all the test items to ensure uniformity and flow of reading has reduced the stress typically caused by these types of flaws as evident in medical student feedback.

The majority of the respondents (n=21) reported use of the NBME item writing guide to facilitate review of their assessments. While the NBME guide is a comprehensive document that details several methods to avoid issues such as construct-irrelevant variance, there are other issues that may appear in exams that create unnecessary stress to the exam takers. To address these problems, the item review committee from our medical school developed an internal style guide (Supplementary File 2) to direct the faculty writing questions and to guide the committee’s review. The style guide, while maintaining the major directives in the NBME guide, includes pointers for writers to ensure ease of reading and uniformity of the questions. The style guide includes recommendations for uniformity of units, drug names, etc., and emphasizes proper placement and style of tables and figures in the question stem. The brevity of our style guide (11 pages compared to the NBME guide’s 84 pages) allows it to serve as a quick reference. This internal guide was endorsed by the curriculum committee and disseminated to faculty to encourage use and improve test-item quality. The student feedback on assessments suggests a positive response thus far, and our item review committee plans to analyze these data after one full cycle.

Limitations

One limitation to this study was the number of responses. We anticipated a higher response rate because the listserv used for solicitation is widely used by professionals in medical education worldwide. A reason for low participation could be due to individuals choosing not to participate if their institution did not have a formal item review process in place. Related to this may have been a misunderstanding of the phrase “process to review student assessment questions before they are used.” For example, if the faculty member writing the item is responsible for review, would a potential respondent consider that to be something other than a review process and decline to participate in the research? In addition, the administration of the survey coincided with the early stages of the COVID-19 pandemic during which faculty and administrators were occupied with higher priorities.

Future Implications

The current study shows that one third of survey participants reported the existence of a similar committee to ensure exam quality in their institutions ( Table 1). This practice may be in development at other medical schools, and hence we feel it is worthwhile to conduct another study to investigate the function and effectiveness of item review committees. What are best practices for use of such a committee with respect to item writers’ and course directors’ review? What is the appropriate combination of skills needed by members of the committee? It would also be of interest to compare our medical school style guide with the internally developed guides from other institutions in order to identify key components of these documents.

## Conclusion

This study provides valuable information about the practices employed by various medical schools in ensuring assessment quality. The results identified that medical schools are using a wide range of practices to ensure assessment quality. The diversity of item review strategies, from no formal review process to multi-step processes, in combination with a variety of tools to guide their review, highlights the need for medical schools to develop item review processes that reflect their resources, needs, and culture. The survey results will be helpful for institutional authorities planning to adopt new processes to review assessment questions or looking to expand upon current procedures.

## Take Home Messages

• Most participating institutions had a process to review assessment questions before use, which suggests that assessment item review is considered best practice.
• Faculty development on exam item writing improves the process of question creation and exam quality.
• Assign oversight of assessment items to a committee or director to ensure the overall exam quality, particularly in areas such as grammar and formatting.
• Membership on an item review committee should include one or more non-medical educators with grammar and editing skills.
• Use of the NBME item writing guide or an internally developed writing guide is helpful in facilitating review of assessment items.

## Notes On Contributors

**Lori M. DeShetler**, PhD, is the Assistant Dean for Assessment and Accreditation in the Department of Medical Education at The University of Toledo. Dr. DeShetler’s current research interests include assessment, COVID-19 impact on medical students, curriculum mapping, and implications of the opioid crisis on medical education. ORCID ID: https://orcid.org/0000-0002-4566-7111

**Bindu Menon**, PhD, is Assistant Professor in the Department of Medical Education at The University of Toledo. Dr. Menon’s research interests include vertical integration of foundational science elements into clinical years, COVID-19 impact on medical students, cognitive assessment of student mastery in assessments and analysis of NBME subject examination results. ORCID ID: https://orcid.org/0000-0002-4436-8208

**Jolene M. Miller**, MLS, is the Director of the Mulford Health Science Library at The University of Toledo. Ms. Miller serves on the university’s MD Program Item Review Committee. Her research interests are the use of reflective practice by health science librarians and role of emotion regulation in library administrators. ORCID ID: https://orcid.org/0000-0003-4422-2708

## Declarations

The author has declared that there are no conflicts of interest.

## Ethics Statement

This research was reviewed by The University of Toledo Social, Behavioral, and Educational Institutional Review Board and was found that the study did not meet the definition of human subjects’ research as outlined in 45 CFR 46.102(e)(1), and therefore did not require Institutional Review Board oversight or approval.

## External Funding

This article has not had any External Funding
