# Supplementary material for: Questioning the questions: Methods used by medical schools to review internal assessment items
Source: MedEdPublish (2016). 2021 Feb 5;10:37. [Version 1] doi: 10.15694/mep.2021.000037.1 (PMC10939609; doi:10.15694/mep.2021.000037.1)
Supplement: Peer review history

# Reviewer report: Reviewer response for version 1

**DOI:** 10.21956/mep.18904.r27012

**Published:** 2021-05-30

**Authors:** Ken Masters

**Affiliations:** 1 Sultan Qaboos University

This review has been migrated. The reviewer awarded 3 stars out of 5 An interesting study on the methods used by medical schools to review assessment questions to ensure quality.I am pleased that the authors have supplied a copy of their questionnaire and documentation for schools intending to introduce some form of question quality review.For me, perhaps the most eye-opening part of this paper is that there are medical schools that do not have a system to review exam questions. It is pleasing to see that this particular school recognised the need to implement such a process. As the authors note, a weakness of the paper is that many possible participants might not have participated because their institution does not have such a process. So, while the paper does give some insight into those processes followed, it does not give an indication of how widespread they are. It would be useful to have follow-up research that addresses this question.Some other issues:• “A frequency threshold of 15% was utilized for identifying themes in the open-ended responses.” This does appear to be rather arbitrary, and I’m not entirely sure that setting such a threshold has support from the literature.• “from across the United States with two from other countries”. I think it would be a good idea to name those other countries, rather than have them classified as “other”.• It is not clear if the survey form was mailed to the participants (and then returned), or if the participants completed the form online (e.g. through Google forms or similar).I look forward to Version 2 of the paper in which these issues and those raised by the first reviewer are addressed.

Reviewer Expertise:

NA

No decision status is available

# Reviewer report: Reviewer response for version 1

**DOI:** 10.21956/mep.18904.r27011

**Published:** 2021-05-19

**Authors:** Keith Wilson

**Affiliations:** 1 Dalhousie University

This review has been migrated. The reviewer awarded 4 stars out of 5 The present study aimed to ascertain methods in use by medical schools in their vetting of questions used on internal examinations. The authors were exploring options for improving question quality in response to assessment feedback from students. They emphasize the need to develop assessments that assess content rather than influences of construct-irrelevant variance. The study was a survey of medical educators that included those that were involved in assessment. They chose a convenience sample derived from members of the DR-ED listserv. Unfortunately, their response rate was lower than expected and they discussed possible reasons for this. Additionally, it is unclear who were the respondents – e.g. general faculty, committee chairs.The questionnaire was brief and explored whether institutions had a process to review questions before they appeared on an examination. It would have been helpful to know whether these same institutions had a post-exam review and how they incorporated a quality improvement cycle. The authors performed a qualitative analysis of the open-ended responses and coded these although it is unclear from the text what method of qualitative analysis was used.Respondents highlighted that much of the onus for content/accuracy fell to the question writers themselves and/or course/unit directors. The authors noted that faculty development would be key to ensuring consistency between writers. Personally, I think a more systematic approach is warranted and indeed some of the respondents had processes in place that involved centralized oversight/quality measures.Despite the lower-than-expected response rate, this is a good article for those that are embarking on improving their test banks. The authors make the case for a more systematic approach in reviewing test items. Although the respondents were mostly from US schools, the concepts translate well to medical schools around the world. Additionally, they included in a second supplementary file their summarized recommendations for question writing: although there are specifics to their school, this resource could be adapted to suit other institutions as it contains many helpful tips.

Reviewer Expertise:

NA

No decision status is available
